# 1222. Impact of Microbiology Reporting Changes in Treatment of AmpC Bloodstream Infections

**DOI:** 10.1093/ofid/ofad500.1062

**Published:** 2023-11-27

**Authors:** Alec Wesolowski, Jessica Miller, Jill Argotsinger, Victoria Gavaghan, Jennifer Dela-Pena

**Affiliations:** Dell Children's Medical Center, Austin, Texas; Advocate Lutheran General Hospital, Chicago, Illinois; Advocate Lutheran General Hospital, Chicago, Illinois; Advocate Lutheran General Hospital, Chicago, Illinois; Advocate Lutheran General Hospital, Chicago, Illinois

## Abstract

**Background:**

Recent literature suggests that AmpC production may be overcalled in Enterobacterales, leading to unnecessary broad-spectrum antimicrobial utilization. The Infectious Diseases Society of America (IDSA) has since reassessed classification of high and low risk organisms with inducible AmpC expression. With this updated guidance, our health system approved changes to our microbiology reporting of AmpC producers in an effort to guide prescribing of appropriate antibiotics. The primary objective of this project was to compare antibiotic prescribing patterns for high and low risk AmpC producers in patients with bloodstream infections (BSI) based on the addition of appended comments to antimicrobial susceptibility results.

**Methods:**

This multicenter, retrospective, cohort study included patients with BSI from October 2022 to March 2023who received at least 48 hours of antimicrobial therapy for the following high and low risk AmpC producers: *Citrobacter* spp*., Enterobacter* spp*., Klebsiella aerogenes, Serratia* spp*., Morganella* spp*., Providencia* spp. The primary endpoint was the number of patients placed on optimal therapy based on adherence to the appended microbiology comment and IDSA guidance. Secondary endpoints included time to effective treatment, time to optimal treatment, duration of therapy, 30 day mortality, microbiological failure, microbiological relapse, 30 day readmission, length of stay, and hospital acquired *Clostridioides difficile* infection.

**Results:**

A total of 191 positive blood cultures were identified with 93 patients (pre-appended comment = 57; post-appended comment = 36) meeting inclusion criteria. Patients were more likely to receive optimal therapy post-appended comment based on IDSA guidance for BSI (38.6% vs 72.2%, OR 4.14; 1.68-10.21). When stratified by high risk and low risk organism, the number of patients on optimal therapy increased in the post-appended comment group but was not statistically significant (high risk: 70.3% vs 88%, OR 3.08; 0.72-13.32; low risk: 10% vs 36.3%, 5.14; 0.92-28.50). No difference in secondary outcomes were detected.

**Conclusion:**

Implementation of an appended microbiology note has the potential to have a positive impact on antimicrobial prescribing patterns in treatment of AmpC bloodstream infections.

**Disclosures:**

**All Authors**: No reported disclosures

